# Mechanisms of resistance against allylamine and azole antifungals in *Trichophyton*: A renewed call for innovative molecular diagnostics in susceptibility testing

**DOI:** 10.1371/journal.ppat.1012913

**Published:** 2025-02-11

**Authors:** Aditya K. Gupta, Tong Wang, Avantika Mann, Vincent Piguet, Anuradha Chowdhary, Wayne L. Bakotic

**Affiliations:** 1 Division of Dermatology, Department of Medicine, Temerty Faculty of Medicine, University of Toronto, Toronto, Ontario, Canada; 2 Mediprobe Research Inc, London, Ontario, Canada; 3 Division of Dermatology, Department of Medicine, Women’s College Hospital, Toronto, Ontario, Canada; 4 Medical Mycology Unit, Department of Microbiology, Vallabhbhai Patel Chest Institute, University of Delhi, Delhi, India; 5 National Reference Laboratory for Antimicrobial Resistance in Fungal Pathogens, Vallabhbhai Patel Chest Institute, University of Delhi, Delhi, India; 6 Bako Diagnostics, Alpharetta, Georgia, United States of America; University of Maryland, Baltimore, UNITED STATES OF AMERICA

## Abstract

The emergence of antifungal resistance calls for continued research efforts to better guide healthcare providers in treatment selection and outcomes. Unlike bacterial infections, treatment of superficial fungal infections is mainly limited to allylamines (terbinafine) and azoles (itraconazole). Here, we aim to update our current understanding of resistance mechanisms against allylamine and azole antifungals in the *Trichophyton* genus. Resistance development has been demonstrated in vitro by challenging *Trichophyton* isolates with allylamines or azoles at levels below the minimum inhibitory concentration (MIC), which corroborates the observation of clinical resistance. Frequently reported mechanisms of resistance include: (I) Alterations of the drug target by single-nucleotide variations (SNVs) of the *SQLE/ERG1* and *ERG11* genes; in particular, *SQLE* SNVs (Leu393Phe, Leu393Ser, and Phe397Leu) have been frequently reported in isolates with high terbinafine MICs; (II) overexpression of the target enzyme for azoles (*ERG11*) and downstream genes in the ergosterol biosynthesis pathway can decrease the effective drug concentration as well as prevent the depletion of ergosterol and the accumulation of toxic sterol intermediates; (III) the up-regulation of drug efflux channels—belonging to the ABC superfamily (*PDR1*, *MDR2*, *MDR3*, *MDR4*), MFS superfamily (*MFS1*), or Pma1 (plasma membrane ATPase 1)—can reduce the effective concentrations of terbinafine and azoles. The possibility of multidrug resistance has been shown in *Trichophyton* strains, of both human and animal origins, harboring multiple resistance mechanisms (e.g., target alteration/overexpression and drug efflux channels). Tackling the issue of antifungal resistance will require an integrated approach with multidisciplinary efforts including surveillance initiatives and antifungal stewardship programs. However, these efforts are hampered by the current limited accessibility of antifungal susceptibility testing as well as the limited choice of antifungals available in routine practice. A better understanding of resistance mechanisms could help develop targeted, molecular-based assays.

## Author summary

Fungal infections of the skin, hair, and nails affect up to one quarter of the world’s population. Unlike treatments for bacterial infections, the number of options for the treatment of superficial fungal infections is more limited, which typically belongs to 2 types of drugs—allylamines (terbinafine) and azoles (itraconazole). In recent years, resistant superficial fungal infections have been increasingly reported globally. However, resistance testing and surveillance have proven to be difficult due to technical limitations. In this article, we aim to update our current understanding of several resistance mechanisms found in superficial fungal infections caused by the *Trichophyton* genus. A better understanding of these mechanisms can aid in the development of advanced molecular diagnostics in place of traditional methods. Common resistance mechanisms identified to date include alterations and overexpressions of the drug target, as well as efflux pumps that reduces the effective drug concentration inside the cell. Resistance can develop due to insufficient drug dosages; once developed, these resistant strains may spread through travel and person-to-person contact. Furthermore, certain wild and domestic animals may carry resistant strains.

## Introduction

**Fig 1:**
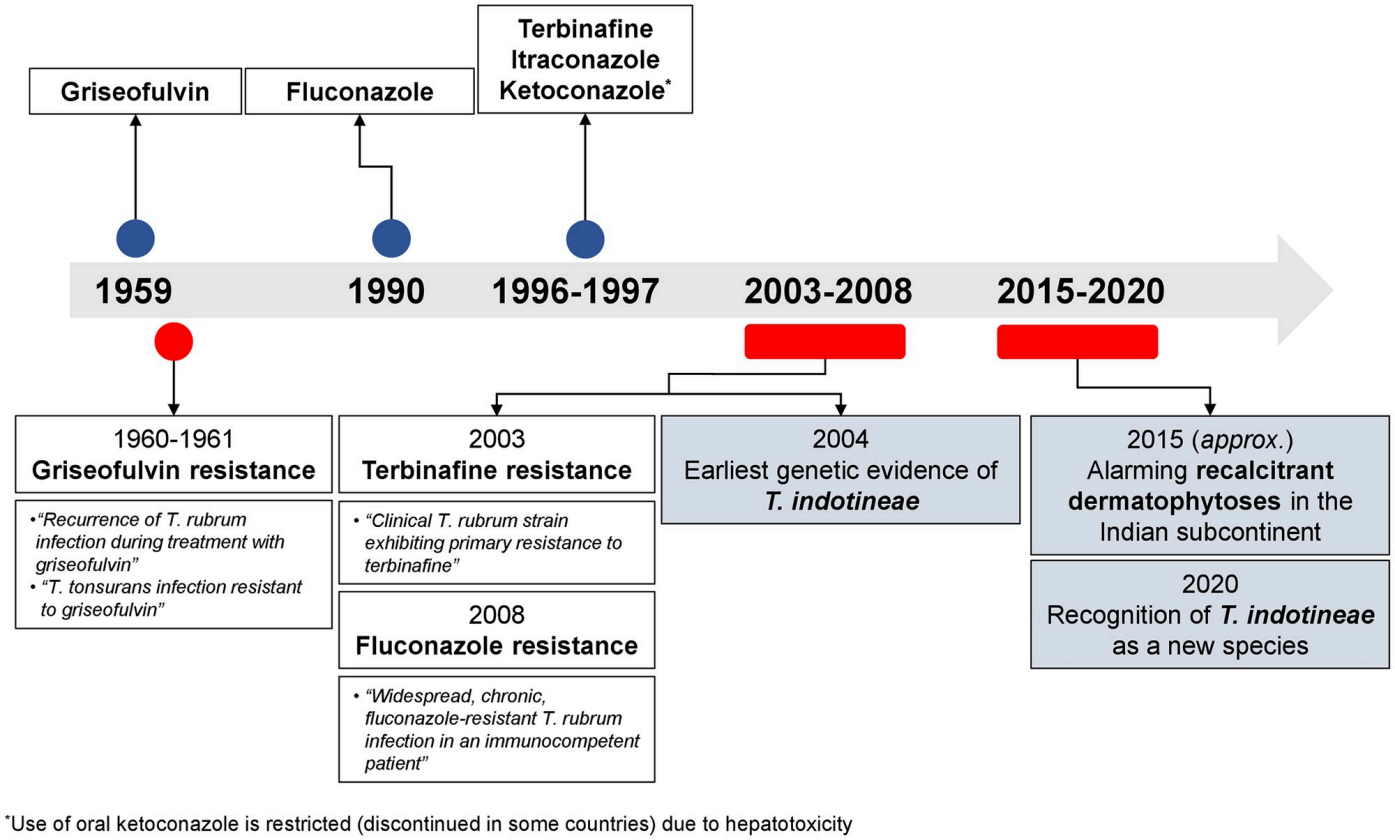
Timeline of oral antifungal developments and resistance reporting for the management of dermatophytosis [3–9].

Treatment failure is not uncommon when it comes to the management of dermatophytosis. Beginning with the introduction of griseofulvin in 1959 (Fig 1), the field of antifungal resistance research has seen an upsurge in recent years due to the waning effectiveness of terbinafine, the most commonly prescribed and cheapest antifungal globally [1,2]. The rising number of cases resistant to terbinafine have been reported globally, and the worldwide spread has been attributed to an outbreak of recalcitrant dermatophytoses in the Indian subcontinent due to *Trichophyton indotineae* [2].

To optimize treatment selection, antifungal susceptibility testing (AFST) has been introduced, and standardized protocols were established by the Clinical and Laboratory Standards Institutes (CLSI) and the European Committee on Antimicrobial Susceptibility Testing (EUCAST) [10]. Through the determination of the minimum inhibitory concentration (MIC)—in reference to the clinical breakpoint—isolates can be categorized as wild-type or resistant to a particular drug. Based on AFST results, treatment selection can be guided using the 90/60 rule which predicts that approximately 90% of infections by wild-type isolates will respond to therapy while approximately 60% of infections by resistant isolates will respond to therapy [11].

Performing AFST for dermatophytes faces numerous challenges [10], namely, the lack of clinical breakpoints to define resistant strains, reliance on fungal culture with a low recovery rate, high costs and need for specialized laboratory resources. In an attempt to increase the uptake and expand the accessibility of AFST, newer methodologies targeting specific resistance markers, without requiring the growth of isolates in culture, have been introduced [10]. In this review, we aim to summarize currently available literature on the landscape of resistance mechanisms against allylamine and azole antifungals in the *Trichophyton* genus.

### *Trichophyton indotineae* resistant to terbinafine and azoles

The issue of antifungal resistance—particularly against terbinafine—has been brought into focus by the outbreak of a newly discovered *Trichophyton* species, *T*. *indotineae* (formerly *T*. *mentagrophytes* genotype VIII). *T*. *indotineae* infection represents a distinct clinical entity that can present as widespread, pruritic tinea corporis or tinea cruris [2]. Atypical features (e.g., eczema-like, psoriasis-like) can also be observed. Although extensive dermatophytoses were once considered a risk mainly for immunocompromised individuals, this no longer appears to be the case with *T*. *indotineae* infections [12].

A multitude of studies across continents have reported resistance mechanisms in *T*. *indotineae* isolates, which is mostly correlated with in vitro terbinafine resistance and less frequently against azoles [12–17]. However, current available data do not support the notion that *T*. *indotineae* is intrinsically resistant against terbinafine, and the alternative use of azoles (e.g., itraconazole, voriconazole) is complicated by its varied bioavailability and safety concerns [2]. Therefore, it is becoming ever more apparent that AFST should be made easily accessible across different healthcare settings to ensure appropriate treatment selection and reduce patient suffering.

### Mechanisms of resistance in *Trichophyton rubrum* and *Trichophyton mentagrophytes* complex

**Fig 2:**
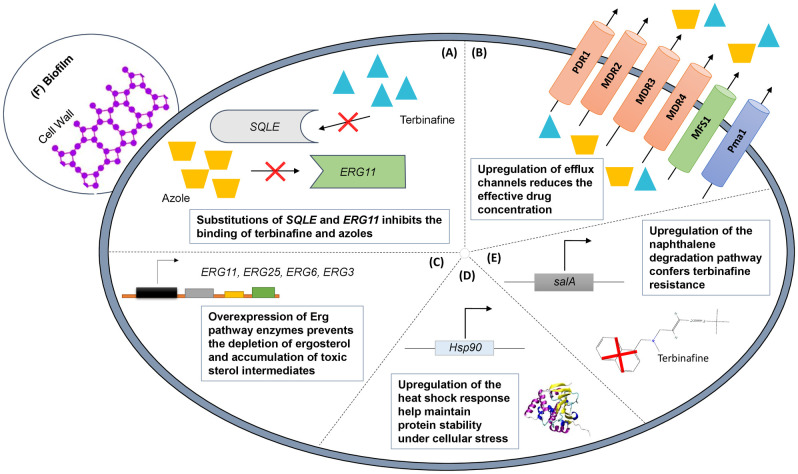
Summary of resistance mechanisms against allylamines and azoles in *Trichophyton*. (A) Single-nucleotide variations of *SQLE* or *ERG11*; (B) up-regulation of drug efflux channels; (C) overexpression of the target enzyme (*ERG11*) for azole antifungals and downstream genes in the ergosterol biosynthesis pathway; (D) up-regulation of the heat shock response (*Hsp90*); (E) overexpression of *salA* as part of the naphthalene degradation pathway; (F) biofilm formation. Three visual elements shown in this figure were modified/reused in accordance with the CC BY 4.0 license (Martinez-Rossi and colleagues [20]), with attribution (2D structure image of terbinafine: PubChem Identifier: 1549008; https://pubchem.ncbi.nlm.nih.gov/compound/1549008#section=Structures), or are in the public domain (https://commons.wikimedia.org/wiki/File:Hsp90.png).

Studies have demonstrated the potential for resistance development when *Trichophyton* isolates are repeatedly challenged with low concentrations of antifungals in vitro; for example, a 2-fold increase in MICs was reported after exposing clinical *T*. *rubrum* isolates to fluconazole and itraconazole [18]. After a period of exposure to increasing concentrations of terbinafine, a wild-type *T*. *rubrum* strain acquired the Phe397Leu substitution in the squalene epoxidase gene (***SQLE/ERG1***) corroborated by an increase in terbinafine MIC from <0.03 to >32 μg/ml [19]. An overview of resistance mechanisms is shown in Fig 2.

Increased expression and posttranscriptional regulation of **drug efflux channels**, belonging to either the ABC (ATP-binding cassette) or MFS (major facilitator superfamily) superfamilies, have been reported in response to terbinafine and azoles [21–30]. **Overexpression of Erg enzymes** (ergosterol biosynthesis pathway) has been implicated to induce resistance—in particular against azoles—by diluting the effective drug concentration [26,28,31–33]. In strains harboring multiple resistance mechanisms (e.g., *SQLE* mutation and up-regulation of drug efflux channels), the possibility for developing **multidrug resistance** has been raised [31,34,35]. Other plausible resistance mechanisms with sparse reports that warrants further clinical validation include biofilms [36], the heat shock response [37], and up-regulation of the naphthalene degradation pathway [38].

### Single-nucleotide variations in the genes encoding squalene epoxidase (*SQLE/ERG1*) and lanosterol 14-α-demethylase (*ERG11*) in *Trichophyton* spp.

Through the induction of enzyme conformational changes that inhibit drug-binding, single-nucleotide variations (SNVs) in the *SQLE* gene have been frequently reported as a mechanism for terbinafine resistance. Phe397Leu is the most common in *T*. *rubrum*, the *T*. *mentagrophytes* complex and *T*. *indotineae* (**Fig A** and **Appendx A and References** in S1 Text). The Ala448Thr substitution thus far has only been reported in the *T*. *mentagrophytes* complex and *T*. *indotineae*; in contrast, substitution at the 440^His^ position is more commonly observed in *T*. *rubrum*.

Based on computational modeling, both 393^Leu^ and 397^Phe^ have been shown to be located adjacent to the terbinafine binding pocket on the squalene epoxidase enzyme [12,13,39,40], this prediction is corroborated by the observation of high terbinafine MICs in strains carrying the Leu393Phe, Leu393Ser, or Phe397Leu substitutions—where almost all strains could be classified as non wild type—confirmed by both CLSI and EUCAST protocols (**Table A** in S1 Text). Furthermore, Leu393Ser has been predicated to significantly alter the SQLE binding affinity to terbinafine [13]. The 448^Ala^ amino acid position, however, is not adjacent to the terbinafine binding pocket and its functional significance warrants further investigation [12,13]. Of interest, a decreased susceptibility to azoles may be associated with the Ala448Thr substitution [12,15,16]. AFST results by both CLSI and EUCAST protocols suggest that strains with the Ala448Thr substitution exhibit high susceptibility to terbinafine (**Table A** in S1 Text).

SNVs in the *ERG11* gene conferring decreased susceptibility to azoles are not as frequently reported. ERG11 in dermatophytes has 2 isoforms, ERG11A and ERG11B. Winter and colleagues reported an *ERG11A* substitution unique to *T*. *quinckeanum* strains with in vitro resistance against fluconazole and itraconazole [41]. No *ERG11A* SNVs have been reported in other *Trichophyton* spp. thus far. However, several *ERG11B* substitutions have been reported in *T*. *indotineae* without a correlation to azole resistance [42,43].

### Overexpression of ergosterol biosynthesis pathway enzymes in *Trichophyton* spp.

As a counter to the depletion of ergosterol and the accumulation of toxic sterol intermediates by allylamines and azoles, the overexpression of target enzymes may serve as a detoxification mechanism. After acquiring itraconazole-resistance in vitro, a *T*. *mentagrophytes* complex strain exhibited an up-regulation of *ERG11* as well as higher quantities of ergosterol compared to a sensitive strain [31]; another study identified the up-regulation of a downstream gene, *ERG25*, in response to clotrimazole [32]. In fluconazole-resistant *T*. *verrucosum* strains of human and animal origins, an increased expression of *ERG11* as well as 2 downstream genes in the ergosterol biosynthesis pathway (*ERG6*, *ERG3*) was found [44].

Recently, an overexpression of *ERG11B* as tandem repeats was discovered by Yamada and colleagues in *T*. *indotineae* strains resistant to itraconazole and voriconazole, while *ERG11A* was overexpressed to a lesser extent [15,16]. An elevated expression of *MDR3* and the presence of *SQLE* SNVs were also found suggesting the possibility of multidrug resistance [15].

### Drug efflux channels in *Trichophyton* spp.

Drug efflux channels—commonly identified as belonging to the ABC or MFS superfamilies in the fungal genome—are integral membrane proteins that mediate the movement of molecules [45]. The ABC-B subfamily (PDR1, MDR2, MDR3, and MDR4) has been associated with multidrug resistance and are abundantly expressed in *Trichophyton* [46]. Exposing clinical *Trichophyton* strains to terbinafine and itraconazole led to a heightened expression of the ABC-B transporters [22].

In a *T*. *rubrum* strain with decreased susceptibility to itraconazole, voriconazole and terbinafine isolated from a tinea corporis patient, Kano and colleagues reported an increased expression of *MDR3* and *MDR4* in tandem with the detection of Leu393Phe in the *SQLE* gene [47]. In another multiresistant *T*. *rubrum* strain, the Phe397Leu substitution in the *SQLE* gene was detected as well as an increase in expression of *MDR2* and *MDR3* [48]; a follow-up study identified *MDR2* as a major contributor to itraconazole resistance [27]. For resistant strains of the *T*. *mentagrophytes* complex, an overall increase in *MDR* expression concurrent with the detection of Phe397Leu in the *SQLE* gene was reported [28]; challenging these strains with itraconazole in vitro led to an increased expression of *PDR1*, *MDR2*, and *MDR3*, while terbinafine increased the expression of *PDR1* [28].

Of the MFS superfamily conferring antifungal resistance, *MFS1* has been implicated in fluconazole and miconazole resistance in the *T*. *mentagrophytes* complex [30]. Another potential drug efflux channel (Pma1; plasma membrane ATPase 1) was recently reported by Ishii and colleagues to play a role in terbinafine resistance using *T*. *rubrum* and may be complementary to the effects of *SQLE* SNVs (Leu393Phe, Phe397Leu) [49].

Besides an elevated level of expression, point mutations and posttranscriptional regulation of transporter genes may increase the functional diversity of the efflux channels, further contributing to resistance development. *PDR1* and *MFS* SNVs were found in a *T*. *mentagrophytes* complex strain from a patient unresponsive to terbinafine and itraconazole [34]. An alternative splicing event of an efflux pump gene, generating an intron-retained isoform, was reported in association with the itraconazole response in *T*. *rubrum* [50].

### Biofilms in *Trichophyton* spp.

The extent of biofilm involvement in the pathogenesis of dermatophytosis is unclear. Biofilms are organized assemblages of microbial communities—sessile and adherent—protected by an extracellular matrix enriched in polysaccharides. When assessing the antifungal susceptibility profile of *Trichophyton* biofilms against planktonic cells or non-biofilm producers in vitro, *Trichophyton* biofilms were less susceptible to terbinafine, itraconazole, econazole, and voriconazole [51–53]. In one study, inhibiting the growth of *Trichophyton* biofilms required itraconazole and voriconazole concentrations at 50 times the MIC than that of planktonic cells [52]. An increase in biofilm thickness—linked to an up-regulation of *ERG25* and *CYP450* genes—was reported by Xiao and colleagues as a response against clotrimazole in the *T*. *mentagrophytes* complex, further highlighting the roles of biofilms in antifungal resistance [32]. In the context of mixed infection, a polymicrobial biofilm composed of *T*. *rubrum* with *Candida albicans* or *C*. *parapsilosis* may be resistant to terbinafine and efinaconazole [54].

In onychomycosis, the secondary development of dermatophytoma (i.e., compacted fungal elements forming a “fungal ball”) complicating treatment is believed to be associated with the formation of biofilms [55]. In 2 patients infected with terbinafine-resistant *T*. *rubrum* (*SQLE* SNV: Leu393Phe) and *T*. *mentagrophytes* var. *interdigitale* (*SQLE* SNV: Phe397Leu), the presence of dermatophytoma was evidenced by the concentrated quantities of hyphae and arthroconidia as well as extracellular polysaccharides [36,56]. Besides reducing drug penetration, studies of *C*. *albicans* biofilms show an up-regulation of drug efflux channels as well as the transition into a state of metabolic quiescence (i.e., dormant cells) that further contributes to antifungal resistance [57,58].

## Conclusions

In recent years, the issue of terbinafine resistance in *Trichophyton* has evolved from sporadic incidents into a matter of public health concern [59,60]. The cause for resistance development may be subtherapeutic dosing, evidenced by the observation of in vitro resistance development when *Trichophyton* spp. are repeatedly challenged with terbinafine or azoles at sub-MIC levels [18,19]. Compounding the evolution of resistance, the spread of these resistant strains could be linked to international travel [61], person-to-person contact [62], and a congregate living setting [63]. This situation is reminiscent of the intercontinental spread of *C*. *auris* [60], suggesting that the global spread to resistant *Trichophyton* species is a distinct possibility.

Tackling these issues will require ongoing surveillance efforts and implementation of antifungal stewardship programs, which is often met with technical and resource limitations in the case of AFST [10]. Our review of the current literature shows multifaceted molecular resistance mechanisms against commonly used antifungals in *Trichophyton* spp., most notably are the target enzyme alterations and overexpression as well as drug efflux channels. As our knowledge on the landscape of antifungal resistance mechanisms expands, the advent of advanced molecular diagnostics may help to increase testing accessibility and capacity.

## Supporting information

S1 Text Appendix A, Fig A, and Table A. (DOCX)
