# Supplementary material for: Mechanisms of resistance against allylamine and azole antifungals in Trichophyton: A renewed call for innovative molecular diagnostics in susceptibility testing
Source: PLoS Pathog. 2025 Feb 11;21(2):e1012913. doi: 10.1371/journal.ppat.1012913 (PMC11813129; doi:10.1371/journal.ppat.1012913)
Supplement: S1 Text — (DOCX) [file ppat.1012913.s001.docx]

**Mechanisms of resistance against allylamine and azole antifungals in *Trichophyton*: A renewed call for innovative molecular diagnostics in susceptibility testing**

Aditya K. Gupta, Tong Wang, Avantika Mann, Vincent Piguet,

Anuradha Chowdhary, Wayne L. Bakotic

*Supporting Information*

*S1 Text*

Table of Contents

[**Appendix A** 2](#_Toc187998451)

[**Fig A** 3](#_Toc187998452)

[**Table A** 4](#_Toc187998453)

# **Appendix A: Literature search**

A literature search was conducted on August 14, 2024 using PubMed, EMBASE (Ovid) and Web of Science. The search/MeSH/Emtree terms used included the following: “Trichophyton”, “mutation”, “drug resistance”, “induced resistance”, “multidrug resistance”. We extracted studies that reported mechanisms of resistance in *Trichophyton* isolates against allylamine and azole antifungals; matching AFST results were extracted if available. Studies that reported AFST results only, contained duplicate datasets, or in case where the full-text was not retrievable, were excluded. Due to evidence of extensive gene transfer and an unclear delineation between species, the term “*T. mentagrophytes* complex” will be used to describe *T. mentagrophytes*, *T. interdigitale*, *T. benhamiae* and *T. tonsurans* [1]; *T. indotineae* will be discussed as a separate species due it being a distinct clinical entity. MIC values were analyzed based on the epidemiolocal cut-off values as reported in the literature [2,3].


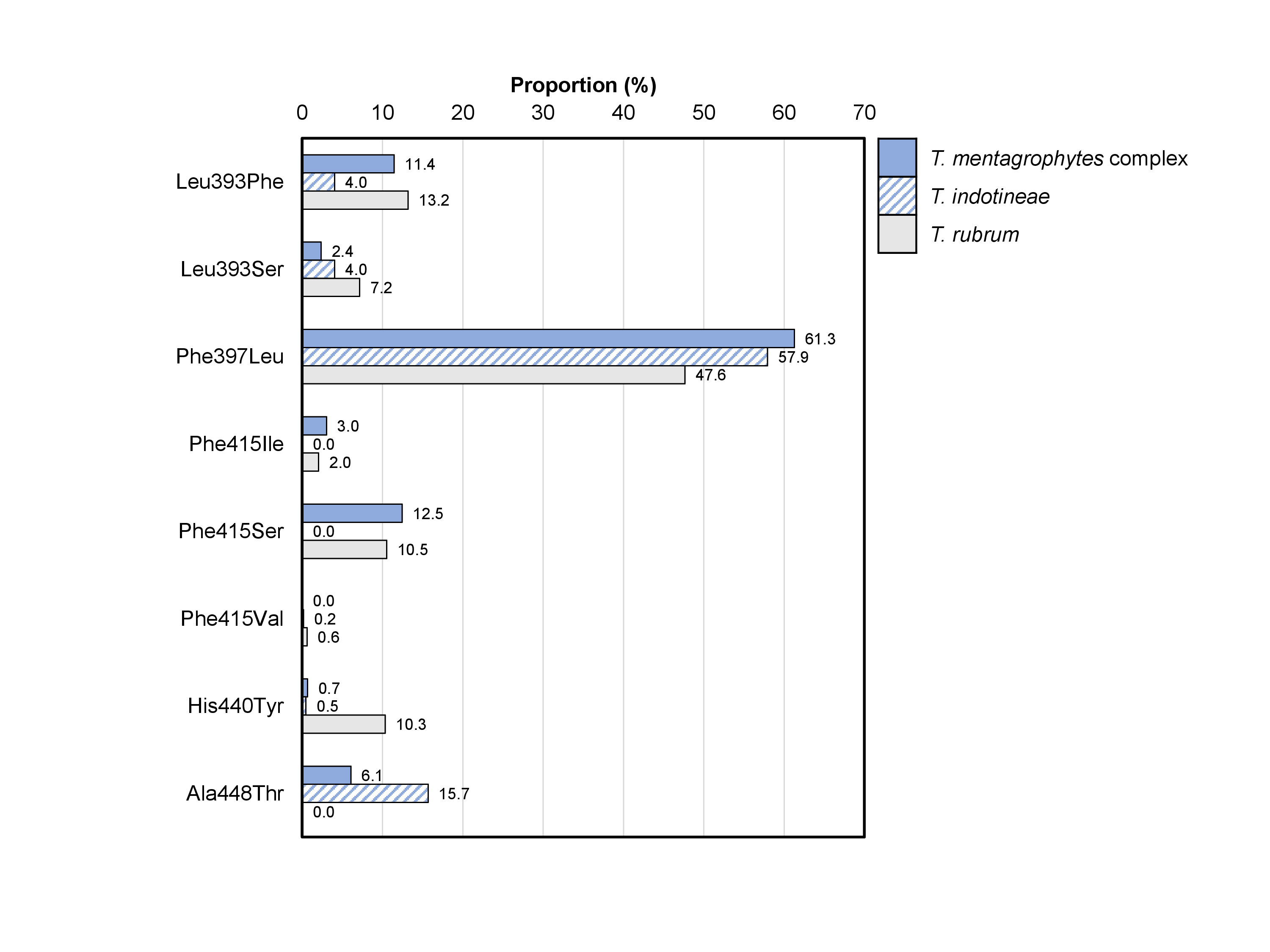


**Fig A.** Across the identified studies [2,4–74], frequencies of commonly detected *SQLE* SNVs in *T. rubrum* (N=978), *T. mentagrophytes* complex (N=297) and *T.* *indotineae* (N=644) are presented.

# **Table A.** *In vitro* susceptibility to terbinafine in *T. indotineae*, the *T. mentagrophytes* complex and *T. rubrum* harbouring *SQLE* mutations.

| **Parameter** | **Leu393Phe** | **Leu393Ser** | **Phe397Leu** | **His440Tyr** | **Ala448Thr** | |
| --- | --- | --- | --- | --- | --- | --- |
| *T. indotineae* | | | | | | |
| CLSI MICs  N  Median  Range  Non-WT^*^, % (N) | 8  16  16-32  100 (8) | 12  0.5  0.5-4  100 (12) | 253  8  0.2-128  99.6 (252) | -  -  -  - | 69  0.125  0.0039-8  1.4 (1) | |
| EUCAST MICs  N  Median  Range  Non-WT^**^, % (N) | 10  16  0.032-64  90 (9) | 12  2  0.25-4  100 (12) | 61  16  0.016-16  93.4 (57) | -  -  -  - | 28  0.0625  0.014-16  14.3 (4) | |
| *T. mentagrophytes* complex | | | | | | |
| CLSI MICs  N  Median  Range  Non-WT^*^, % (N) | 12  32  2-32  100 (12) | 4  0.375  0.125-0.5  75 (3) | 54  8  0.5-32  100 (54) | -  -  -  - | -  -  -  - | |
| EUCAST MICs  N  Median  Range  Non-WT^**^, % (N) | -  -  -  - | -  -  -  - | 4  4  4-8  100 (4) | -  -  -  - | -  -  -  - | |
| *T. rubrum* | | | | | | |
| CLSI MICs  N  Median  Range  Non-WT^*^, % (N) | 47  32  3.2-128  100 (47) | 6  0.25  0.25-1.6  100 (6) | 23  8  2-32  100 (23) | 5  0.015  0.004-0.1  0 | -  -  -  - | |
| EUCAST MICs  N  Median  Range  Non-WT^**^, % (N) | 4  4  0.25-8  100 (4) | 9  0.5  0.25-1  100 (9) | 18  4  0.5-8  100 (18) | -  -  -  - | -  -  -  - | |
| MIC, minimum inhibitory concentration; WT, wild-type  ^*^Non-WT *Trichophyton* spp. isolates are defined as terbinafine MICs ≥0.25 µg/ml according to the Ebert (2020) study [14].  ^**^Non-WT isolates are defined as terbinafine MICs >0.125 µg/ml for the *T. mentagrophytes* complex and MICs >0.03 µg/ml for *T. rubrum*, as per the EUCAST guideline [40].  Note: For MIC values reported as range, only the lowest estimate was used wherever possible. See **S1-S2 Appendix** for literature search method and list of identified studies. | | | | | |  |

# **References**

1. Švarcová M, Větrovský T, Kolařík M, Hubka V. Defining the relationship between phylogeny, clinical manifestation, and phenotype for Trichophyton mentagrophytes/interdigitale complex; a literature review and taxonomic recommendations. Med Mycol. 2023;61. doi:10.1093/mmy/myad042

2. Ebert A, Monod M, Salamin K, Burmester A, Uhrlaß S, Wiegand C, et al. Alarming India-wide phenomenon of antifungal resistance in dermatophytes: A multicentre study. Mycoses. 2020;63: 717–728. doi:10.1111/myc.13091

3. European Committee on Antimicrobial Susceptibility Testing. Overview of antifungal ECOFFs and clinical breakpoints for yeasts, moulds and dermatophytes using the EUCAST E.Def 7.4, E.Def 9.4 and E.Def 11.0 procedures. 2023 [cited 25 Sep 2024]. Available: https://www.eucast.org/astoffungi/clinicalbreakpointsforantifungals

4. Tamimi P, Fattahi M, Ghaderi A, Firooz A, Shirvani F, Alkhen A, et al. Terbinafine-resistant T. indotineae due to F397L/L393S or F397L/L393F mutation among corticoid-related tinea incognita patients. J Dtsch Dermatol Ges. 2024;22: 922–934. doi:10.1111/ddg.15440

5. De Paepe R, Normand AC, Uhrlaß S, Nenoff P, Piarroux R, Packeu A. Resistance Profile, Terbinafine Resistance Screening and MALDI-TOF MS Identification of the Emerging Pathogen Trichophyton indotineae. Mycopathologia. 2024;189: 1–11. doi:10.1007/s11046-024-00835-4

6. Xie W, Kong X, Zheng H, Mei H, Ge N, Hu S, et al. Rapid emergence of recalcitrant dermatophytosis caused by a cluster of multidrug-resistant Trichophyton indotineae in China. Br J Dermatol. 2024;190: 585–587. doi:10.1093/bjd/ljae009

7. Cañete-Gibas CF, Mele J, Patterson HP, Sanders CJ, Ferrer D, Garcia V, et al. Terbinafine-Resistant Dermatophytes and the Presence of Trichophyton indotineae in North America. J Clin Microbiol. 2023;61. doi:10.1128/jcm.00562-23

8. Oladzad V, Nasrollahi Omran A, Haghani I, Nabili M, Seyedmousavi S, Hedayati MT. Multi-drug resistance Trichophyton indotineae in a stray dog. Res Vet Sci. 2024;166: 1–5. doi:10.1016/j.rvsc.2023.105105

9. Noguchi H, Matsumoto T, Kubo M, Kimura U, Hiruma M, Yaguchi T, et al. Dermatophytoma caused by terbinafine-resistant Trichophyton rubrum treated with fosravuconazole. J Dermatol. 2022;49: e407–e408. doi:10.1111/1346-8138.16480

10. Kano R, Nojo H, Noguchi H. Genomic Analysis of Antifungal Drug Resistance Induced in Trichophyton rubrum After Prolonged Culture with Terbinafine. Mycopathologia. 2023;188: 1079–1083. doi:10.1007/s11046-023-00795-1

11. Abastabar M, Babaei M, Mohammadi R, Valadan R, Javidnia J, Zaedi A, et al. Iranian National Survey on Tinea Capitis: Antifungal Susceptibility Profile, Epidemiological Characteristics, and Report of Two Strains with a Novel Mutation in SQLE Gene with Homology Modeling. Mycopathologia. 2023;188: 449–460. doi:10.1007/s11046-022-00657-2

12. Blanchard G, Amarov B, Fratti M, Salamin K, Bontems O, Chang Y-T, et al. Reliable and rapid identification of terbinafine resistance in dermatophytic nail and skin infections. J Eur Acad Dermatol Venereol. 2023. doi:10.1111/jdv.19253

13. Kong X, Song G, Mei H, Zheng H, Tang C, de Hoog S, et al. The Domestic Isolation of Terbinafine- and Itraconazole-Resistant Trichophyton indotineae in Chinese Mainland. Mycopathologia. 2023. doi:10.1007/s11046-023-00761-x

14. Hiruma J, Noguchi H, Shimizu T, Hiruma M, Harada K, Kano R. Epidemiological study of antifungal-resistant dermatophytes isolated from Japanese patients. J Dermatol. 2023;50: 1068–1071. doi:10.1111/1346-8138.16780

15. Gupta AK, Wang T, Polla Ravi S, Cooper EA, Bamimore MA, Lincoln SA, et al. Potential emergence of terbinafine resistance by squalene epoxidase gene mutations: An 18-month cohort study of onychomycosis patients in the United States. Mycoses. 2024;67: 1–9. doi:10.1111/myc.13768

16. Dashti Y, Alobaid K, Al-Rashidi S, Dashti M, AbdulMoneim MH, Al-Enezi M, et al. Autochthonous case of Trichophyton indotineae in Kuwait. J Med Mycol. 2023;33. doi:10.1016/j.mycmed.2023.101432

17. Bortoluzzi P, Prigitano A, Sechi A, Boneschi V, Germiniasi F, Esposto MC, et al. Report of terbinafine resistant Trichophyton spp. in Italy: Clinical presentations, molecular identification, antifungal susceptibility testing and mutations in the squalene epoxidase gene. Mycoses. 2023;66: 680–687. doi:10.1111/myc.13597

18. Bidaud AL, Moreno-Sabater A, Normand AC, Cremer G, Foulet F, Brun S, et al. Evaluation of Gradient Concentration Strips for Detection of Terbinafine Resistance in Trichophyton spp. Antimicrob Agents Chemother. 2023;67: 1–6. doi:10.1128/aac.01716-22

19. Durdu M, Kandemir H, Karakoyun AS, Ilkit M, Tang C, de Hoog S. First Terbinafine-Resistant Trichophyton indotineae Isolates with Phe397Leu and/or Thr414His Mutations in Turkey. Mycopathologia. 2023;188: 2. doi:10.1007/s11046-023-00708-2

20. Jia S, Long X, Hu W, Zhu J, Jiang Y, Ahmed S, et al. The epidemic of the multiresistant dermatophyte Trichophyton indotineae has reached China. Front Immunol. 2023;13: 1113065. doi:10.3389/fimmu.2022.1113065

21. Hiruma J, Kimura U, Noguchi H, Hiruma M, Harada K, Kano R. In vitro Azole Susceptibility Testing of Japanese Isolates of Terbinafine-Resistant Trichophyton indotineae and Trichophyton rubrum. Med Mycol J. 2023;64: 23–25. doi:10.3314/mmj.22-00021

22. Taghipour S, Shamsizadeh F, Pchelin IM, Rezaei-Matehhkolaei A, Mahmoudabadi AZ, Valadan R, et al. Emergence of Terbinafine Resistant Trichophyton mentagrophytes in Iran, Harboring Mutations in the Squalene Epoxidase (SQLE) Gene. Infect Drug Resist. 2020;13: 845–850. doi:10.2147/IDR.S246025

23. Kano R, Kimura U, Noguchi H, Hiruma M. Clinical Isolate of a Multi-Antifungal-Resistant Trichophyton rubrum. Antimicrob Agents Chemother. 2022;66. doi:10.1128/aac.02393-21

24. Dellière S, Joannard B, Benderdouche M, Mingui A, Gits-Muselli M, Hamane S, et al. Emergence of Diffi cult-to-Treat Tinea Corporis Caused by Trichophyton mentagrophytes Complex Isolates, Paris, France. Emerg Infect Dis. 2022;28: 224–228. doi:10.3201/eid2801.210810

25. Kano R, Noguchi H, Hiruma M. A deletion mutation in the amino acid sequence of squalene epoxidase in terbinafine-resistant Trichophyton rubrum. J Infect Chemother. 2022;28. doi:10.1016/j.jiac.2022.02.010

26. Haghani I, Babaie M, Hoseinnejad A, Rezaei-Matehkolaei A, Mofarrah R, Yahyazadeh Z, et al. High Prevalence of Terbinafine Resistance Among Trichophyton mentagrophytes/T. interdigitale Species Complex, a Cross-Sectional Study from 2021 to 2022 in Northern Parts of Iran. Mycopathologia. 2024;189. doi:10.1007/s11046-024-00855-0

27. Kumar P, Das S, Tigga R, Pandey R, Bhattacharya SN, Taneja B. Whole genome sequences of two Trichophyton indotineae clinical isolates from India emerging as threats during therapeutic treatment of dermatophytosis. 3 Biotech. 2021;11: 402. doi:10.1007/s13205-021-02950-1

28. Gnat S, Łagowski D, Dyląg M, Nowakiewicz A. European Hedgehogs (Erinaceus europaeus L.) as a Reservoir of Dermatophytes in Poland. Microb Ecol. 2022;84: 363–375. doi:10.1007/s00248-021-01866-w

29. Burmester A, Hipler UC, Elsner P, Wiegand C. Point mutations in the squalene epoxidase erg1 and sterol 14-α demethylase erg11 gene of T indotineae isolates indicate that the resistant mutant strains evolved independently. Mycoses. 2022;65: 97–102. doi:10.1111/myc.13393

30. Pashootan N, Shams-Ghahfarokhi M, Chaichi Nusrati A, Salehi Z, Asmar M, Razzaghi-Abyaneh M. Phylogeny, Antifungal Susceptibility, and Point Mutations of SQLE Gene in Major Pathogenic Dermatophytes Isolated From Clinical Dermatophytosis. Front Cell Infect Microbiol. 2022;12: 1–10. doi:10.3389/fcimb.2022.851769

31. Bidaud AL, Schwarz P, Chowdhary A, Dannaoui E. In Vitro Antifungal Combination of Terbinafine with Itraconazole against Isolates of Trichophyton Species. Antimicrob Agents Chemother. 2022;66. doi:10.1128/AAC.01449-21

32. Kong X, Tang C, Singh A, Ahmed SA, Al-Hatmi AMS, Chowdhary A, et al. Antifungal Susceptibility and Mutations in the Squalene Epoxidase Gene in Dermatophytes of the Trichophyton mentagrophytes Species Complex. Antimicrob Agents Chemother. 2021;65: e0005621. doi:10.1128/AAC.00056-21

33. Sardana K, Gupta A, Sadhasivam S, Gautam RK, Khurana A, Saini S, et al. Checkerboard Analysis To Evaluate Synergistic Combinations of Existing Antifungal Drugs and Propylene Glycol Monocaprylate in Isolates from Recalcitrant Tinea Corporis and Cruris Patients Harboring Squalene Epoxidase Gene Mutation. Antimicrob Agents Chemother. 2021;65: e0032121. doi:10.1128/AAC.00321-21

34. Noguchi H, Matsumoto T, Hiruma M, Kimura U, Kashiwada-Nakamura K, Kubo M, et al. Cluster infection caused by a terbinafine-resistant dermatophyte at a group home: The first case series in Japan. Acta Derm Venereol. 2021;101: 10–12. doi:10.2340/00015555-3926

35. Yamada T, Maeda M, Alshahni MM, Tanaka R, Yaguchi T, Bontems O, et al. Terbinafine resistance of Trichophyton clinical isolates caused by specific point mutations in the squalene epoxidase gene. Antimicrob Agents Chemother. 2017;61: 1–13. doi:10.1128/AAC.00115-17

36. Yamada T, Yaguchi T, Tamura T, Pich C, Salamin K, Feuermann M, et al. Itraconazole resistance of Trichophyton rubrum mediated by the ABC transporter TruMDR2. Mycoses. 2021;64: 936–946. doi:10.1111/MYC.13286

37. Mohammadi LZ, Shams-Ghahfarokhi M, Salehi Z, Razzaghi-Abyaneh M. Increased terbinafine resistance among clinical genotypes of Trichophyton mentagrophytes/T. interdigitale species complex harboring squalene epoxidase gene mutations. J Med Mycol. 2024;34. doi:10.1016/j.mycmed.2024.101495

38. Monod M, Feuermann M, Salamin K, Fratti M, Makino M, Alshahni MM, et al. Trichophyton rubrum Azole Resistance Mediated by a New ABC Transporter, TruMDR3. Antimicrob Agents Chemother. 2019;63: 1–19. doi:10.1128/AAC.00863-19

39. Gawaz A, Nenoff P, Uhrlaß S, Schaller M. [Treatment of a terbinafine-resistant trichophyton mentagrophytes type VIII]. Hautarzt. 2021;72: 900–904. doi:10.1007/s00105-021-04857-7

40. Singh A, Singh P, Dingemans G, Meis JF, Chowdhary A. Evaluation of DermaGenius® resistance real-time polymerase chain reaction for rapid detection of terbinafine-resistant Trichophyton species. Mycoses. 2021;64: 721–726. doi:10.1111/myc.13271

41. Sacheli R, Harag S, Dehavay F, Evrard S, Rousseaux D, Adjetey A, et al. Belgian national survey on tinea capitis: Epidemiological considerations and highlight of terbinafine-resistant T. mentagrophytes with a mutation on SQLE gene. J Fungi. 2020;6. doi:10.3390/jof6040195

42. Hiruma J, Noguchi H, Hase M, Tokuhisa Y, Shimizu T, Ogawa T, et al. Epidemiological study of terbinafine-resistant dermatophytes isolated from Japanese patients. J Dermatol. 2021;48: 564–567. doi:10.1111/1346-8138.15745

43. Moreno-Sabater A, Normand A-C, Bidaud A-L, Cremer G, Foulet F, Brun S, et al. Terbinafine Resistance in Dermatophytes: A French Multicenter Prospective Study. J fungi (Basel, Switzerland). 2022;8. doi:10.3390/jof8030220

44. Gnat S, Łagowski D, Nowakiewicz A, Dyląg M, Osińska M. Complementary effect of mechanism of multidrug resistance in Trichophyton mentagrophytes isolated from human dermatophytoses of animal origin. Mycoses. 2021;64: 537–549. doi:10.1111/myc.13242

45. Burmester A, Hipler UC, Uhrlaß S, Nenoff P, Singal A, Verma SB, et al. Indian Trichophyton mentagrophytes squalene epoxidase erg1 double mutants show high proportion of combined fluconazole and terbinafine resistance. Mycoses. 2020;63: 1175–1180. doi:10.1111/myc.13150

46. Shankarnarayan SA, Shaw D, Sharma A, Chakrabarti A, Dogra S, Kumaran MS, et al. Rapid detection of terbinafine resistance in Trichophyton species by Amplified refractory mutation system-polymerase chain reaction. Sci Rep. 2020;10: 1–6. doi:10.1038/s41598-020-58187-0

47. Curatolo R, Juricevic N, Leong C, Bosshard PP. Antifungal susceptibility testing of dermatophytes: Development and evaluation of an optimised broth microdilution method. Mycoses. 2021;64: 282–291. doi:10.1111/myc.13202

48. Caplan AS, Todd GC, Zhu YC, Sikora M, Akoh CC, Jakus J, et al. Clinical Course, Antifungal Susceptibility, and Genomic Sequencing of Trichophyton indotineae. JAMA Dermatology. 2024;12208: 1–9. doi:10.1001/jamadermatol.2024.1126

49. Gaurav V, Bhattacharya SN, Sharma N, Datt S, Kumar P, Rai G, et al. Terbinafine resistance in dermatophytes: Time to revisit alternate antifungal therapy. J Med Mycol. 2021;31. doi:10.1016/j.mycmed.2020.101087

50. Shaw D, Singh S, Dogra S, Jayaraman J, Bhat R, Panda S, et al. MIC and Upper Limit of Wild-Type Distribution for 13 Antifungal Agents against a Trichophyton mentagrophytes-Trichophyton interdigitale Complex of Indian Origin. Antimicrob Agents Chemother. 2020;64. doi:10.1128/AAC.01964-19

51. Jabet A, Brun S, Normand AC, Imbert S, Akhoundi M, Dannaoui E, et al. Extensive Dermatophytosis Caused by Terbinafi ne-Resistant Trichophyton indotineae, France. Emerg Infect Dis. 2022;28: 229–233. doi:10.3201/eid2801.210883

52. Khurana A, Masih A, Chowdhary A, Sardana K, Borker S, Gupta A, et al. Correlation of in vitro susceptibility based on MICs and squalene epoxidase mutations with clinical response to terbinafine in patients with TINEa corporis/cruris. Antimicrob Agents Chemother. 2018;62: 1–9. doi:10.1128/AAC.01038-18

53. Kano R, Kimura U, Kakurai M, Hiruma J, Kamata H, Suga Y, et al. Trichophyton indotineae sp. nov.: A New Highly Terbinafine-Resistant Anthropophilic Dermatophyte Species. Mycopathologia. 2020;185: 947–958. doi:10.1007/s11046-020-00455-8

54. Saunte DML, Hare RK, Jørgensen KM, Jørgensen R, Deleuran M, Zachariae CO, et al. Emerging Terbinafine Resistance in Trichophyton: Clinical Characteristics, Squalene Epoxidase Gene Mutations, and a Reliable EUCAST Method for Detection. Antimicrob Agents Chemother. 2019;63: 1–9. doi:10.1128/AAC.01126-19

55. Hsieh A, Quenan S, Riat A, Toutous-Trellu L, Fontao L. A new mutation in the SQLE gene of Trichophyton mentagrophytes associated to terbinafine resistance in a couple with disseminated tinea corporis. J Mycol Med. 2019;29: 352–355. doi:10.1016/j.mycmed.2019.100903

56. Osborne CS, Leitner I, Hofbauer B, Fielding CA, Favre B, Ryder NS. Biological, biochemical, and molecular characterization of a new clinical Trichophyton rubrum isolate resistant to terbinafine. Antimicrob Agents Chemother. 2006;50: 2234–2236. doi:10.1128/AAC.01600-05

57. Nenoff P, Verma SB, Ebert A, Süß A, Fischer E, Auerswald E, et al. Spread of terbinafine-resistant trichophyton mentagrophytes type VIII (India) in Germany–“the tip of the iceberg?” J Fungi. 2020;6: 1–20. doi:10.3390/jof6040207

58. Russo G, Toutous Trellu L, Fontao L, Ninet B. Towards an early clinical and biological resistance detection in dermatophytosis: About 2 cases of Trichophyton indotineae. J Fungi. 2023;9: 1–9. doi:10.3390/jof9070733

59. Uhrlaß S, Mey S, Koch D, Mütze H, Krüger C, Monod M, et al. Dermatophytes and skin dermatophytoses in Southeast Asia—First epidemiological survey from Cambodia. Mycoses. 2024;67: 1–12. doi:10.1111/myc.13718

60. Singh A, Masih A, Monroy-Nieto J, Singh PK, Bowers J, Travis J, et al. A unique multidrug-resistant clonal Trichophyton population distinct from Trichophyton mentagrophytes/Trichophyton interdigitale complex causing an ongoing alarming dermatophytosis outbreak in India: Genomic insights and resistance profile. Fungal Genet Biol. 2019;133. doi:10.1016/j.fgb.2019.103266

61. Siopi M, Efstathiou I, Theodoropoulos K, Pournaras S, Meletiadis J. Molecular Epidemiology and Antifungal Susceptibility of Trichophyton Isolates in Greece: Emergence of Terbinafine-Resistant Trichophytonmentagrophytes Type VIII Locally and Globally. J fungi (Basel, Switzerland). 2021;7. doi:10.3390/jof7060419

62. Astvad KMT, Hare RK, Jørgensen KM, Saunte DML, Thomsen PK, Arendrup MC. Increasing Terbinafine Resistance in Danish Trichophyton Isolates 2019-2020. J fungi (Basel, Switzerland). 2022;8. doi:10.3390/jof8020150

63. Łagowski D, Gnat S, Nowakiewicz A, Osińska M, Dyląg M. Intrinsic resistance to terbinafine among human and animal isolates of Trichophyton mentagrophytes related to amino acid substitution in the squalene epoxidase. Infection. 2020;48: 889–897. doi:10.1007/s15010-020-01498-1

64. Burmester A, Hipler UC, Hensche R, Elsner P, Wiegand C. Point mutations in the squalene epoxidase gene of Indian ITS genotype VIII T. mentagrophytes identified after DNA isolation from infected scales. Med Mycol Case Rep. 2019;26: 23–24. doi:10.1016/j.mmcr.2019.09.001

65. Singh A, Masih A, Khurana A, Singh PK, Gupta M, Hagen F, et al. High terbinafine resistance in Trichophyton interdigitale isolates in Delhi, India harbouring mutations in the squalene epoxidase gene. Mycoses. 2018;61: 477–484. doi:10.1111/MYC.12772

66. Noguchi H, Matsumoto T, Kubo M, Kimura U, Hiruma M, Tanaka M, et al. Effective Response of Dermatophytoma Caused by Terbinafine-Resistant Trichophyton interdigitale Solely to Topical Efinaconazole. Mycopathologia. 2022;187: 421–422. doi:10.1007/s11046-022-00636-7

67. Kitauchi Y, Kumagai Y, Inoue-Masuda Y, Sugiura M, Sato T, Yaguchi T, et al. Tinea corporis caused by terbinafine-resistant Trichophyton rubrum successfully treated with fosravuconazole. J Dermatol. 2021;48: e329–e330. doi:10.1111/1346-8138.15900

68. Gueneau R, Joannard B, Haddad N, Alby F, Jullien V, Schlatter J, et al. Extensive dermatophytosis caused by terbinafine-resistant Trichophyton indotineae, successfully treated with topical voriconazole. Int J Antimicrob Agents. 2022;60. doi:10.1016/j.ijantimicag.2022.106677

69. Suzuki S, Mano Y, Furuya N, Fujitani K. Discovery of terbinafine low susceptibility trichophyton rubrum strain in Japan. Biocontrol Sci. 2018;23: 151–154. doi:10.4265/bio.23.151

70. Yamada T, Maeda M, Nagai H, Salamin K, Chang Y-T, Guenova E, et al. Two different types of tandem sequences mediate the overexpression of TinCYP51B in azole-resistant Trichophyton indotineae. Antimicrob Agents Chemother. 2023;67: e0093323. doi:10.1128/aac.00933-23

71. Rudramurthy SM, Shankarnarayan SA, Dogra S, Shaw D, Mushtaq K. Mutation in the Squalene Epoxidase Gene of Trichophyton. Antimicrob Agents Chemother. 2018;62: e02522-17.

72. Salehi Z, Shams-Ghahfarokhi M, Razzaghi-Abyaneh M. Antifungal drug susceptibility profile of clinically important dermatophytes and determination of point mutations in terbinafine-resistant isolates. Eur J Clin Microbiol Infect Dis. 2018;37: 1841–1846. doi:10.1007/s10096-018-3317-4

73. Yamada T, Yaguchi T, Maeda M, Alshahni MM, Salamin K, Guenova E, et al. Gene Amplification of CYP51B: a New Mechanism of Resistance to Azole Compounds in Trichophyton indotineae. Antimicrob Agents Chemother. 2022;66. doi:10.1128/aac.00059-22

74. Teo JWP, Cheng JWS, Chew KL, Lin RTP. Whole genome characterization of Trichophyton indotineae isolated in Singapore. Med Mycol. 2024;62: 1–4. doi:10.1093/mmy/myae012
